# The International Conference on Intelligent Biology and Medicine (ICIBM) 2018: genomics meets medicine

**DOI:** 10.1186/s12920-018-0448-5

**Published:** 2019-01-31

**Authors:** Degui Zhi, Zhongming Zhao, Fuhai Li, Zhijin Wu, Xiaoming Liu, Kai Wang

**Affiliations:** 1 Center for Precision Health, School of Biomedical Informatics, The University of Texas Health Science Center at Houston, Houston, TX 77030 USA; 2 Department of Biomedical Informatics, Ohio State University, Columbus, OH 43210 USA; 3 Department of Biostatistics, Brown University, Providence, RI 02912 USA; 4 Human Genetics Center, School of Public Health, The University of Texas Health Science Center at Houston, Houston, TX 77030 USA; 5 Raymond G. Perelman Center for Cellular and Molecular Therapeutics, Children’s Hospital of Philadelphia, Philadelphia, PA 19104 USA; 6 Department of Pathology and Laboratory Medicine, University of Pennsylvania, Philadelphia, PA 19104 USA; 7 Present address: College of Public Health, University of South Florida, Tampa, FL 33612 USA

## Abstract

During June 10–12, 2018, the International Conference on Intelligent Biology and Medicine (ICIBM 2018) was held in Los Angeles, California, USA. The conference included 11 scientific sessions, four tutorials, one poster session, four keynote talks and four eminent scholar talks that covered a wide range of topics ranging from 3D genome structure analysis and visualization, next generation sequencing analysis, computational drug discovery, medical informatics, cancer genomics to systems biology. While medical genomics has always been a main theme in ICIBM, this year we for the first time organized the BMC Medical Genomics Supplement for ICIBM. Here, we describe 15 ICIBM papers selected for publishing in *BMC Medical Genomics*.

## Introduction

For the past 6 years, the International Conference on Intelligent Biology and Medicine (ICIBM) meeting has been covering extensive cutting edge research topics in genome and medicine [1–5]. At 2018, with tremendous advancements in computational precision medicine, ICIBM received exceedingly high volume of submission and a good portion of the submissions are focused on genome medicine [6]. We selected 15 high quality papers from ICIBM 2018 meeting covering a range of topics in medical genomics. In these papers, traditional topics in genomics, such as gene regulatory pathway inference and gene signature/set analysis, are covered with a translational twist. More emphases are given to solve real medically relevant questions. Altogether, these papers present various cutting edge researches in medical genomics, suggesting that as a field, medical genomics is playing increasingly important roles in advancing clinical application of bioinformatics and computational genomics. We also see new artificial intelligence algorithms are being applied to biomedical problems. Below, we briefly summarize the main findings from each paper.

## The science program for the ICIBM 2018 medical genomics track

Predicting cellular responses to drugs has been a major challenge for personalized drug therapy regimen. In the first paper by Wang et al. [7], the authors compared pathway activity inference approaches for predicting drug response of cancer cell lines, based on the gene expression and drug response data from Cancer Cell Line Encyclopedia (CCLE). Pathway activities were first inferred from gene expression data and then used to build machine learning models to predict drug response. Their results on all 24 CCLE drugs demonstrated that pathway-based models are more capable of capturing drug-relevant mechanisms than gene-based models, whiling achieving comparable prediction performance. Modeling with inferred pathway activity is promising to predict drug response and provide biological insights into the mechanisms of drug actions.

The next paper by Zhang et al. [8] aimed to develop an lncRNA-related method to identify traditional mRNA biomarkers. Traditional methods do not consider the regulatory and positional relationship between mRNA and lncRNA. The combined analysis of mRNA and lncRNA is likely to facilitate the identification of biomarkers with higher confidence. They selected mRNA biomarkers based on two criteria: 1) differential expression between normal and cancer tissue samples; 2) a positional relationship to lncRNAs that are differentially expressed between normal and cancer samples. Their results suggested that mRNAs expression profiles coexpressed with positionally related lncRNAs can provide important insights into early diagnosis and gene therapy of HCC.

The next paper from Fan et al. [9] proposed a gene signature selection strategy for TCGA data by integrating the gene expression data, the methylation data and the prior knowledge about cancer biomarkers. A fuzzy rule based classification method was applied in the model construction for performance evaluation. The prediction results from the cross validation and independent validation indicated that, the gene signatures extracted with our fuzzy rule based integrative feature selection strategy were more robust, and had the potential to offer better prediction results. Notably, PTCHD3 gene was selected as a discriminating gene in 3 out of the 6 cancers, which suggested that it might play important role in the cancer risk and would be worthy for the intensive investigation.

In the next paper by Djotsa et al. [10], the authors propose a new function prediction based approach to discover cancer driver genes through a gene-based permutation approach. Their method not only covers gene coding regions as many other methods focused on, but also interrogates non-coding regions. The permutation model was implemented independently using seven popular deleteriousness prediction scores covering splicing regions, coding regions and pan-genome. They applied this new approach to somatic single nucleotide variants from whole-genome sequences of 119 breast and 24 lung cancer patients and compared the performance of the seven scores. Their results suggested multiple candidate driver genes, and showed the advantage of using pan-genome deleteriousness prediction scores, compared to using missense-specific deleteriousness prediction scores.

The next study by Han et al. [11] was aimed to identify functional exon skipping events and genetic variation influencing the alternative splicing in Alzheimer’s disease (AD). They analyzed RNA-Seq data of hippocampal tissues. Their RNA-Seq analysis revealed not only two functional exons in *RELN* and one exon in *NOS1* more skipped in AD patients compared to cognitively normal elderly individuals, but also splicing-affecting SNPs associated with amyloid-β deposition in the brain. Their integrative analysis with multiple omics and neuroimaging data confers possible mechanisms for understanding AD pathophysiology through exon skipping. This result may provide a useful resource of a novel therapeutic development.

In the next paper by Menor et al. [12], a novel method for discovering somatic mutation based prognostic signatures for cancer was demonstrated and evaluated. The proposed mutation tumor frequency ratio (MFR) profiles used the log2 ratio of the tumor mutation frequency to the paired normal mutation frequency of a gene. Prognostic signatures for lung adenocarcinoma and colorectal adenocarcinoma were generated using Cox analysis of MFR and other existing types of somatic mutation profiles. Among all methods tested, only MFR profiles achieves statistically significant risk stratification on the validation dataset. This result demonstrated the robustness of MFR profiles and its potential to be a powerful prognostic tool in cancer.

Although many methods have been developed for predicting the single nucleotide variant effects, only a few have been specifically designed for identifying deleterious sSNVs (synonymous single nucleotide variants). In the next work by Shi et al. [13], the authors proposed a method, namely IDSV (Identification of Deleterious Synonymous Variants), to predict deleterious sSNVs by using a wide variety of features. Experimental results on benchmark datasets demonstrated that IDSV outperforms other methods in identifying pathogenic sSNVs. Their results indicate that besides splicing and conservation features, a new translation efficiency feature is also an informative feature. While the function regions annotation and sequence features were weakly informative, they may have the ability to detect deleterious sSNVs when combined with other features.

The objective of Cheng et al. [14] was to utilize publicly available data sets to identify potential predictive copy number variation (CNV) biomarkers of chemotherapeutic response in pediatric sarcomas. 206 CNV profiles derived from pediatric sarcoma biopsies collected from the public databases TARGET and NCBI-Gene Expression Omnibus (GEO) were compared against that of 22,255 healthy individuals called from the Database of Genomic Variants (DGV) and a pool of 63 genes that harbored amplifications and/or deletions that were found frequently associated with recurrence across all three sarcoma types. By integrating CNVs of Cancer Cell Line Encyclopedia (CCLE) identified in the pool of 63 genes with drug-response data, 33 CNVs were identified as potential predictive biomarkers of therapeutic response. These CNV signatures could potentially be used to delineate patient populations that respond versus those that do not respond to a particular chemotherapy. The large-scale analyses of CNV-drug screening provides a platform to evaluate genetic alterations across aggressive pediatric sarcomas and provides novel insights into the potential prognostic as well as predictive biomarkers of therapeutic response.

Haplotype phasing is important in cancer genomics, as it facilitates a comprehensive understanding of clonal architecture and further provides potentially valuable reference in clinical diagnosis and treatment. In the next paper of this supplement, Wang et al. [15] proposed a graph-based computational pipeline to reconstruct clonal haplotypes directly from cancer sequencing data. Comparing to the existing approaches, the proposed algorithm reduces the computation complexity by three bounding strategies. According to a series of experiments, the proposed algorithm was able to identify about 90% in average of the preset clonal haplotypes under different simulation configurations. Therefore, it is considered as a practical algorithm and is robust when the mutation rates are low.

The paper by Li et al. [16] represents the first regulatory network analysis of genes associated with cleft lip (CL), one of the most frequently occurred congenital birth defects in humans. The authors identified two types of regulation pairs, transcription factor (TF)-gene pair and microRNA-gene pair, using manually curated CL genes and regulatory databases, and constructed comprehensive miRNA-TF mediated co-regulatory networks specific for human CL. They reported novel pathways with potential association with CL etiology, as well as critical hub miRNAs, TFs and genes that may have important roles in the regulation process of CL. Their analysis revealed that the CL-specific regulatory networks had critical disease-causing miRNAs. This study not only unveiled novel miRNAs for further experimental design but also provided some insight into regulatory mechanisms of human CL.

The next paper by Chen and Xu [17] integrated several types of networks to explore association between food metabolites and Alzheimer’s Disease (AD). They systematically investigated the role of food-derived metabolites and constructed a context-sensitive gene-metabolite-food network to integrate heterogeneous chemical and genetic information. Using this network, they modeled context-specific inter-relationships among foods, metabolites, human genes and AD. Their results showed that top-ranked food metabolites were specifically enriched in herbs and spices and shared many common pathways with AD, including the amyloid processing pathway. This study represents the first systems approach to characterizing the effects of food-derived metabolites in AD pathogenesis by mining the relationships among foods, metabolites and human genes.

The paper by Chiu et al. [18] presents a Deep Neural Network (DNN) model for learning data embeddings of high-dimensional mutation and gene expression profiles. Based on this model, prediction of drug response of cancer cell lines and tumors based on this model to a panel of 265 anti-cancer drugs outperformed two classical machine learning methods and four analog DNN models. Results from analysis of PanCanAtlas (CITE) data confirmed known molecular mechanisms underlying the resistance of chemotherapy and identified a novel agent, CX-5461, with anti-cancer potential in treating gliomas and hematopoietic malignancies.

Xia et al. [19] present a method, VirTect, for detecting virus integration sites simultaneously using sequencing data from multiple related samples, from different locations or different time points in the same patient. VirTect uses joint analysis of short reads spanning breakpoints of integration sites from multiple samples. Using a local, precise sandwich alignment algorithm, VirTect achieved high specificity and breakpoint accuracy compared to alternative methods, with lower computational time and memory requirement. With joint analysis of multiple sample data without pooling, VirTect gave exactly the same breakpoint estimate for shared integration sites among different samples, providing convenient input for subsequent analysis of tumor heterogeneity and evolution.

During the past 11 years, genome-wide association studies have reported many thousands of association signals between genetic variants and a specific phenotype. Phenome-Wide Association Studies (PheWAS) take advantage of large patients-based cohorts with a panel of wide range of phenotypes and are well suited to facilitate new marker SNPs as well as SNPs with pleiotropy. The paper by Zhao et al. [20] presents a PheWAS study considering 67 traits on a large African American cohort and provides invaluable information on this often under-studied population. Their results validated 29 known associations, including eight that were reported for the first time in African Americans. The cross-race validation of disease associated genetic variants strengthens the evidence of these loci’s involvement in the disease process and may help identify genes in the causal pathway.

RNA-sequencing has now become a routine technique in genomic studies and data continue to accumulate with increasing rate in the public domain. This enables repurposing of existing data for new applications. In the final paper of this Supplement, Zeng et al. [21] presented a deep learning approach that selects normal tissue samples to serve as reference for those cancer studies that do not have own reference samples. The results benchmarked by TCGA data demonstrate its potential for tapping samples from external sources as reference samples for such cancer studies. This could boost the sample size of normal references for cancer (or other disease) studies with few or no normal sample included in the studies. As the public data depositories continue to grow, methods like this will have increasing utility in practice.
